# Insulator Protein Helps Organize the Gaps in the Axon's Insulation

**DOI:** 10.1371/journal.pbio.1002259

**Published:** 2015-09-25

**Authors:** Richard Robinson

**Affiliations:** Freelance Science Writer, Sherborn, Massachusetts, United States of America

## Abstract

The protein P0 has long been known to play a crucial role in holding together the myelin sheath that insulates peripheral nerves. A new study reveals that P0 is also important for organizing the nodes of Ranvier that occupy the gaps in the insulation. Read the Research Article.

Schwann cells wrap around and insulate the axons of peripheral neurons, leaving exposed only the tiny uninsulated gaps known as the nodes of Ranvier. The combination of tight insulation and widely spaced gaps allows the action potential to jump from node to node, speeding conduction. On either side of the node is the so-called paranode, a highly organized region in which a set of proteins form junctions between the axon and the lateral edges of the multiple layers of the Schwann cell’s plasma membrane. Further away yet is another specialized region, the juxtaparanode, with its own distinct set of proteins, followed by the internode.

The layers of insulation in the internodes are held together largely through the homophilic adhesion of myelin protein zero (P0), and it has been widely thought that P0 was restricted to the internodal regions. But in a new study in *PLOS Biology*, Valérie Brügger, Claire Jacob, and colleagues show that P0 plays a critical role in organizing the paranodes and nodes, that it depends on histone deacetylases to do so, and that some mutations in P0 that are responsible for a form of human peripheral neuropathy maintain the protein’s homophilic adhesion but disrupt its paranodal and nodal interactions [1].

From their previous work, the authors knew that histone deacetylases 1 and 2 (HDAC1 and HDAC2) played a role in Schwann cell development, so here they asked what their function might be in the adult Schwann cell. To do so, they created a line of inducible single and double knockout mice, allowing them to switch off production in adult mice. They found that mice without either protein developed both motor and sensory disturbances, accompanied by a dramatic reduction in expression of P0.

The most striking effect of loss of the deacetylases was on the structure of the nodes and paranodes. At the paranode, several proteins interact to form septate junctions, important in maintaining the integrity of the paranodal boundaries and preventing invasion of juxtaparanodal proteins. Loss of HDAC1 and 2 and the consequent loss of P0 altered the location and stability of two junction proteins, called neurofascin 155 and Caspr. A voltage-gated K+ channel that is normally restricted to the juxtaparanode was mislocalized to the paranodes. A nodal protein, neurofascin 186, was largely absent, and the nodes of Ranvier were wider than normal.

These effects could be mitigated by transducing Schwann cells in tissue culture with a lentivirus that supplied P0. Unexpectedly, the authors found P0 in the paranodes and nodes of Ranvier, and they showed it interacted directly with both neurofascin 155 and neurofascin 186, indicating it is part of both the paranodal and nodal complexes (Fig 1).

**Fig 1:**
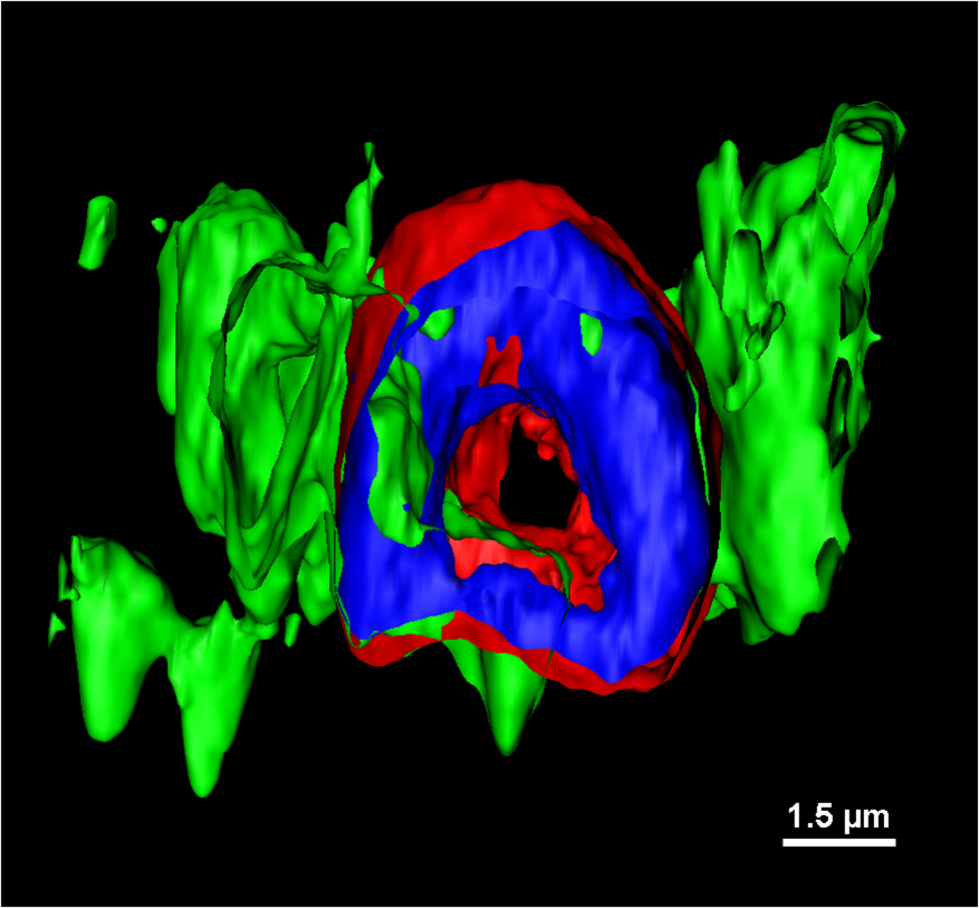
P0 (in green) colocalizes with neurofascins (155 in red and 186 in blue and red) in the paranode and node of Ranvier, forming a ring around the axon (empty space in the middle). Credit: Valérie Brügger, Boris Egger, and Claire Jacob

Charcot-Marie-Tooth disease (CMT) is a group of peripheral neuropathies characterized by demyelination, altered axon–Schwann cell interactions, or both. Mutation in P0 is one cause of the disease. To test the relevance of their newly discovered function of P0 to CMT, the authors examined several disease-causing mutants for their ability to bind to the neurofascins (all the tested mutants were still able to bind other P0’s). They found that one mutant that causes a demyelinating form of CMT retained its ability to bind to both neurofascins. In contrast, three mutants that cause an adult-onset altered-interaction form were unable to bind to neurofascin 155, and two of the three also had reduced ability to bind to neurofascin 186. Unlike wild-type P0, exogenously supplying these mutant proteins causing adult-onset disease could not rescue the effects of HDAC 1 and 2 in tissue culture, indicating the importance of the binding of P0 to the neurofascins for maintenance of proper morphology and function of the paranodes and nodes of Ranvier.

These results identify an important new function for P0 and deepen the understanding of how the complex nodal structure of the peripheral myelinated axon is maintained. They also open up new avenues for understanding the molecular mechanisms underpinning some forms of CMT. There are more than 80 genes known to cause CMT, with more yet to be found. It will be interesting to see if mutations in HDAC1 or 2 may be responsible for some cases of the disease.

## Abbreviations

CMT: Charcot-Marie-Tooth
P0: protein zero
